# The decisional balance, attitudes, and practice behaviors, its predicting factors, and related experiences of advance care planning in Taiwanese patients with advanced cancer

**DOI:** 10.1186/s12904-022-01073-5

**Published:** 2022-11-02

**Authors:** Yueh-Chun Chen, Hsiang-Ping Huang, Tao-Hsin Tung, Ming-Yang Lee, Randal D. Beaton, Yung-Chang Lin, Sui-Whi Jane

**Affiliations:** 1 Department of Nursing, Ditmanson Medical Foundation Chia-Yi Christian Hospital, Chia-Yi, Taiwan; 2 Department of Nursing, Chang Gung University of Science and Technology, Taoyuan City, Taiwan; 3 Evidence-based Medicine Center, Taizhou Hospital of Zhejiang Province affiliated to Wenzhou Medical University, Linhai, Zhejiang China; 4 Division of Hematology/Oncology, Department of Internal Medicine, Ditmanson Medical Foundation Chia-Yi Christian Hospital, Chia-Yi, Taiwan; 5 Psychosocial & Community Health and Health Services, Schools of Nursing and Public Health, University of Washington, Seattle, USA; 6 Division of Hematology/Oncology, Department of Internal Medicine, Chang Gung Memorial Hospital, Lin-Ko, Taiwan; 7 Dean of Academic Affairs, Department of Nursing, College of Nursing, Chang Gung University of Science and Technology, 261, Wen-Hua 1st Rd., Gui-Shan Dist, 33303 Tao-Yuan City, Taiwan

**Keywords:** Advanced cancer, Advance care planning, Decisional balance, Attitude, Practice behaviors, Qualitative responses

## Abstract

**Background:**

Patients with advanced cancer are prone to experience burdensome physical, psychological, and financial consequences. Healthcare providers may not fully appreciate advanced cancer patients’ medical care autonomy, such as at that emboded by Advance Care Planning (ACP), and by doing so may compromise their quality of end-of-life (EOL). Hence, it is essential for healthcare providers to effectively assess and communicate with patients’ regarding their medical decisions before their patients are incapacitated by their disease progression. The purpose of this investigation was to describe the decisional balance, attitudes, and practice behaviors of ACP and its predictors of ACP-related experiences in Taiwanese patients with advanced cancer.

**Methods:**

This cross-sectional, descriptive study employed a mixed-methodsquantitative and qualitative design with a sample of 166 patients that were purposely recruited from in-patient oncology units at a regional teaching hospital in southern Taiwan. Study data consisted of patient replies to a 34-item self-report tool, Decisional Balance, Attitudes, Practice Behaviors of ACP (DAP-ACP) and 4 semi-structured questions.

**Result:**

Findings indicated that, in general, study participants exhibited favorable ACP-decisional balance and positive ACP-attitudes & practice behaviors. The results also indicated that gender, educational level, and cancer diagnosis were associated with significant differences on the “ACP-decisional balance” and “ACP-attitudes” scales. In addition, our findings documented that the participants’ gender and educational level were significant predictors of both ACP-decisional balance and ACP-attitudes. Furthermore the participants’ ACP-practice behaviors were predicted by ACP-decisional balance, but not with their ACP-attitudes. The qualitative analysis of the semi-structured questions identified six themes in responses to current medical decision making (e.g., compliance with physician instructions, family engagement in treatment decision-making); and eight themes pertaining to future ACP-related concerns were identified (e.g., family conflict, effectiveness of time-limited trials).

**Conclusion:**

To promote patients’ engagement in ACP, the healthcare professional need to assess and advocate patients’ concerns or attitudes regarding ACP in a timely manner. In addition, factors or concerns that might influence patients’ responses to ACP derived from both the quantitative and qualitative findings of this current study need to be considered especially in initiating the dialogue regarding ACP with patients with advanced cancer.

**Trial registration:**

No. CYCH 2,019,072, Date of registration 5 Dec 2019.

**Supplementary Information:**

The online version contains supplementary material available at 10.1186/s12904-022-01073-5.

## Background

Cancer is one of the leading causes of death worldwide, accounting for approximately 10 million deaths in 2020[1]. Along with the progression of their cancer(s), patients and their caregivers often encounter burdensome physical, psychological, and financial consequences, compromising their quality of life (QOL) and/or their quality of end-of-life (EOL). According to the principle(s) of palliative care (PC), one of the most important elements of ethical and legal considerations for palliative care is advance care planning (ACP)[2], encompassing Advance Directives (AD), Health Care Agent (HCA), and medical decisions for EOL (i.e., Do Not Resuscitate, DNR; Physician Orders for Life-Sustaining Treatment, POLST)[3]. Hence, the provision of an adequate quality of EOL care, including respect for cancer patients’ autonomy regarding medical decisions in light of their impending incompetency, is an important challenge and task for patients, as well as their families and perhaps especially for the front-line healthcare providers.

Existing research investigations have documented that the fulfillment of patients’ EOL wishes resulted in the partial amelioration of patients’ anxiety/depression as well as the degree of emotional burden for family decision-makers [4] as served to deter conflicts amongst patients and their family members, and healthcare providers [5], and also reduced medical costs [6]. However, in Europe less than 10% of patients with advanced cancer discussed ACP with their healthcare providers [7]. This percentage was 33.3% in the US [8], but only 16.1% in Taiwan [9]. Similarly, studies conducted in Hong Kong found that less than 20% of nursing home residents (*n* = 238) ever discussed ACP issues with their family members or healthcare professional [10] and for seriously ill patients and their caregivers still perceived limited autonomy in ACP decision-making and lack of readiness and awareness of discussion of ACP [11]. Further, a meta-analysis of 38 studies [12] found that an average of 33–38% of EOL patients actually received non-beneficial treatment (NBT) during their last 6 months of life. For instance, 19.5% of cancer patients received chemotherapy (C/T) during their last month of life in France [13] and this percentage was 9.8% in Italy [14]. From the perspective of quality of PC, such NBT not only contributed to their unnecessary suffering, but also resulted in costly low-value healthcare utilization [15] with 40% of cancer treatment costs arising from their care during the last month of life [16]. While 80% of US cancer patients expressed a desire to be informed about screening harms, only 9.5% of patients were ever informed about the risks of over-treatment by their physicians [17].

To date, ACP-related studies primarily have focused on the end-stage renal disease [18], chronic pulmonary disease [19], dementia [20], and incurable cancer in western countries [21]. Considering the nature of health care systems, patients’ degree of expressed autonomy about medical decision-makings, and patient-physician communication patterns in the Asia-Pacific region, such as Taiwan, findings of investigations of patients from Western countries may not generalize and fully reflect the ACP phenomena in Taiwan. With an emphasis on protecting patients’ medical decisions, Taiwan was the first Asian country to endorse the “Patient Right to Autonomy Act” in 2020 [22].Thus, it is imperative for healthcare providers to explore what extent of ACP are embraced by Taiwanese advanced cancer patients, how they perceive and respond to ACP, and what factors influencing their ACP decision making from the perspective of trans-cultural care. Hence, the purposes of this mixed methods quantitative and qualitative investigation were to describe the decisional balance, attitudes, and practice behaviors, and their ACP correlates in Taiwanese patients with advanced cancer.

## Materials and methods

### Sample and study design

This cross-sectional descriptive study employed a mixed methods --qualitative and quantitative—designs and explored the decisional balance, attitudes, and practice behaviors of ACP in Taiwanese patients with advanced cancer and their experiences and preparations with current treatment decision-making and future ACP. The participants were purposely sampled from a regional teaching hospital in southern Taiwan from January to June 2020. Participants were included if they met the following criteria: age ≥ 20 years; ability to understand, speak, and read Chinese; diagnosed with stage III–IV cancer and admitted for cancer-related treatment. Patients were excluded from study if they had received hospice care (Taiwanese patients must agree with and sign ACP-related document prior to transferring to hospice care, which could affects patients’ responses to ACP); and/or any other physical symptom (e.g., severe pain or dyspnea or deteriating cognition) or psychological conditions (i.e., severe anxiety or derepssive mood) which might have prevented them from participating in the research protocol.

The sample size was determined based on an effect size of 0.13 [23], alpha = 0.05 with a power of 90% [24], accounting for a total of 166 participants. To achieve the qualitative data saturation, an estimated 15-20% of the 166 participants were invited to take part in an additional face-to-face interview.

This study was approved by the institutional review board from Ditmanson Medical Foundation Chia-Yi Christian Hospital, Taiwan (IRB # 2,019,072). Participants who met the inclusion criteria were approached and provided with an explanation of the research purposes and procedures and their rights associated with study participation when they were admitted hospital for their cancer-related treatments. After obtaining written consent, the research associate provided participants with a battery of questionnaires and unsealed envelopes and participants were asked to place their completed questionnaires in the sealed envelopes. The majority of participants (*n* = 145, 87% ) completed questionnaires on their own with an average of 33 min and 32 invited participants were interviewed with each semi-structured interview lasting an average of additional 26 min.

### Instrument

#### Demographic and medical information

The demographic information gathered from each participant included their age, gender, religion, marital status, and education level as well as medical information including their cancer diagnosis, stage, and previous/current treatments.

#### Decisional balance, attitudes, practice behaviors of ACP (DAP-ACP)

The decisional balance (12 items), attitudes (7 items), and practice behaviors (15 items) of ACP were assessed with a 34-item self-report tool *(DAP-ACP)*, which was originally developed by Fried et al. [25], Decisional Balance, Beliefs, Processes of Changes Survey in Advance Care Planning. This instrument was based on Transtheoretical Model (TTM) of behavior change and in prior studies with cancer patients demonstrated good factor loadings (> 0.5) and internal consistency (Cronbach’s α = 0.76–0.93). The premise(s) of the TTM is that individuals progress through five (5) stages as they prepare for and actually modify their behaviors. These five (5) stages include: pre-contemplation (no current intention to change behavior), contemplation (thinking about changing behavior), preparation (commitment to changing behavior soon), action (signifying a recent change in behavior), and maintenance (ongoing behavior change). Based upon the TTM [26], Fried et al. [25] developed the *DAP-ACP* tool, encompassing decisional balance (representing an individual’s evaluation of the pros and cons of changing their behavior), values/beliefs (attitude, values and religious beliefs and medical misconceptions serving as potential barriers to participation), and, finally, the processes of change of ACP (behavioral and cognitive processes used to foster behavioral change). According to the constructs of behavioral change process, decisional balance not only facilitates individuals’ values/beliefs (attitudes) in an effort to address issues that serve as potential barriers to, and facilitators of behavior change, but also predict the ensuing behavioral changes [25, 27]. Thus, the authors in this current study proposed that both decisional balance and values/beliefs (attitudes) of ACP would correlate with the behavioral practices of ACP.

In 2014, Sudore et al. employed the 34-item *DAP/ACP* instrument (Cronbach’s α = 0.87–0.93) to assess the effectiveness of a web-based intervention designed to enhance older adults willingness to engage in ACP decision-making. Participant replies on all decisional balance, attitudes, and practice of behavior sub-scales rated with a 5-point Likert scale (5 *strongly agree*, 1 *strongly disagree*) (a rating of 3 (neutral) was used as a cut-off point in this study); a high score indicated positive intentions to engage ACP. Condiering this current study with an tans-cultural dessign in nature, the authors translated each item into Chinese and then back translated into English to ensure the cultural coherence of the original and translated versions of this tool. In addition, the validity of DAP-ACP was separately examined with both the exploratory factor analyses (EFA) and the confirmatory factor analyses (CFA).

### EFA

The 12 items ACP-decisional balance were included in a principal components analysis (PCA) and suggested two factors, the Pros and Cons, indicating that all item loadings were > 0.40, accounting for 51.4% of the total item variance. The 17 items ACP-attitude were included in PCA and all item loadings were > 0.40, accounting for 52.5% of the total item variance. Also, the 15 items ACP-attitude were included in PCA and suggested three factors and all item loadings were > 0.40, accounting for 59.8% of the total item variance (Appendix 1). Thus, the results of the EFA indicated good factor structure and construct validity of the DAP-ACP with our patient population.

### CFA

The final items identified in the EFA were included in CFA. The fit for the ACP-decisional balance measure was excellent fit, with goodness of fit index (GFI) = 0.935, adjusted goodness of fit index (AGFI) = 0.903, *X*^2^/df = 1.364, and root mean square error of approximation (RMSEA) = 0.047. The correlation between the factors of ACP-decisional balance in the confirmatory sample was *r* = − 0.08. For the ACP-attitude and ACP-practice scales also indicated good for excellent fit, with GFI = 0.963, AGFI = 0.914, *X*^2^/df = 1.860, and RMSEA = 0.072 ; GFI = 0.904, AGFI = 0.864, *X*^2^/df = 1.696, and RMSEA = 0.065 (r = 0.26), respectively. The results of the CFA indicated good to excellent fit construct validity of the DAP-ACP in this current study.

#### Qualitative interview of ACP-related decision-making and preparation

The semi-structured interview guide was originally adapted from McMahan et al. [28] that explored what steps best prepare cancer patients and their surrogates for decision making of ACP (i.e., have you ever made an important or significant medical decision about serious illness; have you ever talked with someone else about death and dying). Cognizant of the conservative nature of communication pattern of Taiwanese patients (Taiwanese tended to be reluctant to self-disclise details of life expeiences), we refined queries specific to their life expericnes or current treatments, allowing them easily comprehended, such as “What decisions have you made to treat cancer?”, “What was the most difficult decision you made?”; “If you can prepare for a critical treatment decision in advance, what would you do?”; “What is the most challenging part (for you) when making such a treatment decision?”.

### Data analyses

The SPSS 20.0 was employed for descriptive (i.e., percentage, mean, Standard Deviation, SD) and inferential analyses (i.e., independent *t* test, one-way analysis of variance, ANOVA, Scheffe’s comparisons). Pearson correlation coefficient was used to examine the inter correlations among ACP decisional balance, attitudes, and practice behaviors scales and multiple regression analyses were used to examine their correlation with the DAP/ACP scales, The qualitative responses were analyzed with “The Five Steps of Qualitative Data Analysis: Climbing Up a Ladder of Abstraction” proposed by Carney [29], including coding and labeling the responses, identifying themes, exploring the major descriptive themes, outlining deeper construction, and fitting the descriptive responses. The 15-20% of the 166 participants (*n* = 32) were invited for further qualitavie interviews until the saturation of the qualitative responses was achieved. To ensure the reliability of the qualitative analyses, the first and corresponding authors initially analyzed the first five and following ten participants’ responses independently, then any discrepancy was further clarified with the inter-rater agreement of the qualitative responses of 87.6% and 84.3%, respectively. Lastly, the first author analyzed the remaining patients’ responses, then the corresponding author re-confirmed the entire analyses. In addition, the rigor of qualitative analyses were further confirmed based on the transfer-ability, dependability and confirm-ability as suggested by Lincoln and Guba [30].

## Results

### Sample characteristics

Among the 275 patients who were deemed eligible for study participation, 109 were declined to participate and the remaining 166 patients consented (acceptance rate 60.4%). The study participant sample had a mean age of 55.9 years and the majority were married (66.3%), had completed high school (36.7%) and were currently unemployed (65.1%). The vast majority of study participants (81.4%) reported that treatment decisions were made by patients themselves. In terms of the clinical conditions of the participants, most of them were diagnosed with colon rectum cancer (19.9%); followed by breast cancer (18.1%) and head and neck cancer (11.4%) (Table 1).

**Table 1:** Sample demographic and patient medical characteristics (*n* = 166)

Variable		*f* (%)		Mean ± SD
Gender				
Female		85(51.2)		
Male		81(48.8)		
Age				55.91 ± 10.43
< 45yrs		20(12.0)		
45-59yrs		93(56.0)		
> 60 yrs		53(31.9)		
Marital status				
Single/Divorced		56(33.7)		
Married		110(66.3)		
Education				
Primary/Junior School		54(32.5)		
High School		61(36.7)		
College		24(14.5)		
University or Higher		27(16.2)		
Employment				
Yes		58(34.9)		
No		108(65.1)		
Religion				
Buddhism		41(24.7)		
Taoism		96(57.8)		
Christian		14(8.4)		
None		15(9.0)		
Medical-Decision Made by ^a^ Patient Self Family Members Friends Physician Cancer Diagnoses	135 (81.38) 131 (78.92) 65 (39.16) 113 (68.07)			
Colon & Rectum	33(19.9)			
Lung	12(7.2)			
Breast	30(18.1)			
Liver	9(5.4)			
Head & neck	19(11.4)			
Other	63(38.0)			
Cancer Staging				
III	61(36.7)			
IV	104(62.7)			
Duration of Diagnosis			29.11 ± 32.892	
1–12 months	81(48.8)			
13–24 months	24(14.5)			
> 24 months	61(36.7)			
Current Treatment ^†^				
Chemotherapy	206(142)			
Radiotherapy	24(14.5)			
Surgery	2(1.2)			
Symptom Management	8(4.8)			

Note: ^a^ More than 100%

### Decisional balance, attitudes, practice behaviors of ACP

Employing the cut-off points of 3 for each item of decision balance scales neutral responses (total score 3*12 = 36), when compared to the total ACP score of 42.66, there was a significant difference between items responses (*t* (165) = 90.758, *p* < 0.001) in terms of positive agreement(s). The most positive agreement was reported for the item “I would feel better knowing I have done what I can to plan for my future”, followed by “Understanding my wishes would help my loved ones to ensure I get the care I want”. The item with the strongest level of disagreement was “It would be hard to do ACP because I don’t like thinking about being very ill “, which is considered a neutral responses ACP decisional balance (*p* = 0.537), and the others were in significant positive agreement with ACP decisional balance (Table 2).

**Table 2:** The descriptive data associated with each item of decisional balance, attitudes, practice behaviors of ACP (*n* = 166)

Variable	Strongly Agree	Agree	Neutral	Disagree	Strongly Disagree			
***Decisional Balance Subscale*** **(12 items)**	*f* (%)	*f* (%)	*f* (%)	*f* (%)	*f* (%)	M ± SD	*t*	*p*
1. It would be hard to do Advance Care Planning (ACP) because I don’t like thinking about being very ill. ^b^	6(3.6)	65(39.2)	27(16.3)	50(30.1)	18(10.8)	3.05 ± 1.13	0.618	0.537
2. Doing ACP would simplify how decisions would be made if I were very ill.	33(19.9)	101(60.8)	14(8.4)	13(7.8)	5(3.0)	3.87 ± 0.93	12.087	< 0.001^***^
3. It would be hard to do ACP because I don’t like thinking about death. ^b^	24(14.5)	59(35.5)	21(12.7)	53(31.9)	9(5.4)	3.22 ± 1.20	2.335	0.021^*^
4. I don’t want to talk with loved ones about end-of-life decisions. ^b^	19(11.4)	77(46.4)	25(15.1)	40(24.1)	5(3.0)	3.39 ± 1.07	4.732	< 0.001^***^
5. Doing ACP would make it easier on my close family and friends.	33(19.9)	105(63.3)	17(10.2)	8(4.8)	3(1.8)	3.95 ± 0.81	15.026	< 0.001^***^
6. It would be hard to do ACP because there are too many options to consider for my end-of-life care. ^b^	5(3.0)	53(31.9)	26(15.7)	69(41.6)	13(7.8)	3.19 ± 1.07	2.328	0.021^*^
7. Understanding my wishes would help my loved ones to ensure I get the care I want.	27(16.3)	117(70.5)	16(9.6)	6(3.6)	0(0)	3.99 ± 0.64	20.098	< 0.001^***^
8. I would feel better knowing I have done what I can to plan for my future.	35(21.1)	109(65.7)	11(6.6)	10(6.0)	1(0.6)	4.01 ± 0.76	17.083	< 0.001^***^
9. ACP would go against my lifestyle of living in the moment. ^b^	2(1.2)	30(18.1)	20(12.0)	91(54.8)	23(13.9)	3.62 ± 0.98	8.193	< 0.001^***^
10. Doing ACP would give me peace of mind.	31(18.7)	111(66.9)	17(10.2)	6(3.6)	1(0.6)	3.99 ± 0.70	18.279	< 0.001^***^
11. ACP would help me to keep control over what happens to me at the end of life.	25(15.1)	112(67.5)	21(12.7)	8(4.8)	0(0)	3.93 ± 0.68	17.482	< 0.001^***^
12. It doesn’t make sense to do ACP because my wishes for my end-of-life care might change. ^b^	31(18.7)	30(18.1)	87(52.4)	18(10.8)	0(0)	3.55 ± 0.92	7.779	< 0.001^***^
***Attitudes Subscale*** **(7 items)**								
13. If you fill out a document such as a living will, the doctors are more likely to stop life support too soon. ^b^	5(3.0)	17(10.2)	51(30.7)	77(46.4)	16(9.6)	3.49 ± 0.91	6.972	< 0.001^***^
14. There is no need for me to do ACP because once you reach a certain age; the doctors aren’t going to use machines to try to keep you alive. ^b^	5(3.0)	20(12.0)	37(22.3)	86(51.8)	18(10.8)	3.55 ± 0.94	7.565	< 0.001^***^
15. There is no need to do ACP because my doctor knows what I want for my end-of-life care. ^b^	5(3.0)	39(23.5)	40(24.1)	67(40.4)	15(9.0)	3.29 ± 1.02	3.648	< 0.001^***^
16. There is no need for me to do ACP because I will always be able to make my own treatment decisions. ^b^	3(1.8)	43(25.9)	34(20.5)	77(46.4)	9(5.4)	3.28 ± 0.97	3.681	< 0.001^***^
17. ACP would interfere with the plans that the Lord has for me. ^b^	1(0.6)	15(9.0)	11(6.6)	110(66.3)	29(17.5)	3.91 ± 0.81	14.510	< 0.001^***^
18. There is no need for me to do ACP because if I am made to suffer, then there must be a good reason for it. ^b^	3(1.8)	38(22.9)	23(13.9)	84(50.6)	18(10.8)	3.46 ± 1.02	5.791	< 0.001^***^
19. Planning for future medical care only makes sense for those who are much older or sicker than I am. ^b^	1(0.6)	36(21.7)	17(10.2)	89(53.6)	23(13.9)	3.58 ± 1.00	7.544	< 0.001^***^
***Practice Behaviors Subscale*** **(15 items)**								
20. I looked for information on ACP.	5(3.0)	43(25.9)	19(11.4)	76(45.8)	23(13.9)	2.58 ± 1.11	-4.836	< 0.001^***^
21. I thought about information people have given me on ACP.	8(4.8)	57(34.3)	18(10.8)	68(41.0)	15(9.0)	2.85 ± 1.14	-1.707	0.090
22. I remembered information people have given me on the need for ACP.	6(3.6)	53(31.9)	20(12.0)	69(41.6)	18(10.8)	2.76 ± 1.12	-2.763	0.006^**^
23. I reviewed my advanced care documents so that I know what they say.	6(3.6)	51(30.7)	16(9.6)	71(42.8)	22(13.3)	2.69 ± 1.15	-3.514	0.001^**^
24. There is someone I can talk to about doing ACP.	17(10.2)	91(54.8)	21(12.7)	31(18.7)	6(3.6)	3.49 ± 1.03	6.207	< 0.001^***^
25. It is important that I make sure people close to me have copies of my advanced care plans with them.	20(12.0)	97(58.4)	25(15.1)	22(13.3)	2(1.2)	3.67 ± 0.90	9.604	< 0.001^***^
26. Now is the right time to do ACP.	21(12.7)	83(50.0)	31(18.7)	27(16.3)	4(2.4)	3.54 ± 1.00	7.067	< 0.001^***^
27. It is important that I make sure that I know where my advanced care documents can be found.	23(13.9)	111(66.9)	20(12.0)	11(6.6)	1(0.6)	3.87 ± 0.75	14.880	< 0.001^***^
28. I can do ACP even if it is difficult for my loved ones.	24(14.5)	101(60.8)	29(17.5)	11(6.6)	1(0.6)	3.82 ± 0.78	13.520	< 0.001^***^
29. Doing ACP makes me feel like a person who cares about my close family and friends.	28(16.9)	112(67.5)	21(12.7)	4(2.4)	1(0.6)	3.98 ± 0.67	18.788	< 0.001^***^
30. I can count on my loved ones to help me with ACP.	7(4.2)	59(35.5)	38(22.9)	55(33.1)	7(4.2)	3.02 ± 1.02	0.306	0.760
31. I think of myself as someone who can reduce suffering for me and my family by doing ACP.	30(18.1)	110(66.3)	19(11.4)	6(3.6)	1(0.6)	3.98 ± 0.71	17.846	< 0.001^***^
32. My loved ones will support me as I do ACP.	18(10.8)	93(56.0)	50(30.1)	5(3.0)	0(0)	3.75 ± 0.69	14.054	< 0.001^***^
33. The thought of having an advanced care plan makes me feel good about taking responsibility for my health care.	33(19.9)	113(68.1)	14(8.4)	6(3.6)	0(0)	4.04 ± 0.66	20.512	< 0.001^***^
34. I feel committed to doing ACP.	32(19.3)	81(48.8)	40(24.1)	12(7.2)	1(0.6)	3.79 ± 0.86	11.843	< 0.001^***^

Note: ^b^ negative frame (keyed inversely) * *p* < 0.05; ** *p* < 0.01; ***p < 0.001

By using the cut-off points of 3 for each item of ACP-attitudes as neutral responses (total score 3*7 = 21) then to compare with the total score of 24.57, these were significantly difference between item reponses (*t* (165) = 57.067, *p* < 0.001). The item with the most positive agreement on the ACP-attitudes scale was “ACP would interfere with the plans that the Lord has for me”, and secondly, “Planning for future medical care only makes sense for those who are much older or sicker than I am”. The item with the lowest level of agreement on this scale was “There is no need for me to do ACP because I will always be able to make my own treatment decisions” (Table 2).

Similarly, using the cut-off of 3 for each item of ACP-practice behaviors as neutral (total score 3*15 = 45), when to compared to the total score of 51.83, there was significantly differences between items reponses (*t* (165) = 78.228, *p* < 0.001). The most positive agreement was for the ACP-practice behavior item: “The thought of having an advanced care plan makes me feel good about taking responsibility for my health care” (*p* < 0.001); the item for which there was the lowest level of agreement was “I looked for information on ACP” (*p* < 0.0001) (Table 2).

### The differences of patients’ characters of ACP and its correlates

The one-way ANOVA was used to examine the differences associated with participants’ characteristics among decisional balance, attitudes, practice behaviors of ACP. Results showed that gender, educational level, and cancer diagnosis variables were all associated with significant differences on the “ACP-decisional balance” and “ACP-attitudes” scales. The ACP-decisional balance and ACP-attitudes of female patients were more likely to endorse “strongly agree” compared to the male subsample and the ACP-decisional balance and ACP-attitudes of those with an “above college” education were significantly more positive; that is, in agreement compared to those who reported their education as “below high school”. Further, participants diagnosed with breast cancer” were more positive on the ACP-Decisional balance and ACP-attitude scale than those diagnosed with “head and neck cancer” (Table 3). Pearson correlation coefficients were generated between all of the ACP scales which documented positive correlations between ACP-decisional balance and ACP-attitudes and ACP-decisional balance and ACP-practice behaviors (both *p* < 0.001), but no statistically significant correlation was uncovered between ACP-attitudes and ACP-practice behaviors (*p* = 0.185).

**Table 3:** Results of statistical tests of participants’ demographic characteristics and their scores on the decisional balance, attitudes, practice behaviors scales of the ACP (*n* = 166)

	Decisional Balance				Attitudes				Practice Behaviors		
Variable	M ± SD	F/*p*	Scheffe’s test		M ± SD	F/*p*	Scheffe’s test		M ± SD	F/*p*	Scheffe’s test
Gender		4.882/0.029^*^				9.180/0.003^**^				0.132/0.717	
Female	43.59 ± 5.56				25.65 ± 4.81				52.05 ± 8.11		
Male	41.68 ± 5.57				23.43 ± 4.60				51.59 ± 8.02		
Age		1.860/0.159				2.226/0.111				0.412/0.663	
a.<45yrs	42.10 ± 7.45				25.85 ± 4.34				51.85 ± 8.32		
b.45-59yrs	43.39 ± 5.07				24.90 ± 4.71				52.28 ± 7.91		
c.>60 yrs	41.58 ± 5.71				23.51 ± 5.07				51.02 ± 8.25		
Marital status		0.251/0.617				3.085/0.081				0.658/0.418	
a.Other	42.96 ± 6.93				25.48 ± 4.94				52.54 ± 8.25		
b.Married	43.43 ± 6.55				24.10 ± 4.72				51.46 ± 7.95		
Education		3.483/0.033^*^				3.321/0.039^*^				1.755/0.176	
a.Primary/high school	41.95 ± 5.34				24.01 ± 4.80				51.06 ± 7.97		
b.Colledge	43.58 ± 5.32				24.96 ± 5.48				53.92 ± 8.93		
c.University or higher	44.89 ± 6.55	0.048^*^	c > a		26.59 ± 3.83	0.042^*^	c > a		53.22 ± 7.24		
Employment		0.029/0.865				2.414/0.122				0.032/0.858	
a.Yes	42.76 ± 5.69				23.78 ± 4.65				51.67 ± 8.74		
b.No	42.60 ± 5.62				24.99 ± 4.88				51.91 ± 7.68		
Religion		1.095/0.337				0.235/0.791				0.393/0.676	
a.Taoism	42.46 ± 5.51				24.46 ± 4.89				51.64 ± 8.23		
b.Christian	44.79 ± 5.56				24.79 ± 5.21				53.64 ± 6.77		
c.None	42.47 ± 6.68				25.33 ± 3.98				51.87 ± 7.63		
Cancer diagnosis		2.612/0.027^*^				4.217/0.001^**^				1.829/0.110	
a.Colon & rectum	42.06 ± 5.87				24.82 ± 3.75				49.45 ± 7.40		
b.Lung	41.42 ± 6.19				22.83 ± 5.01				54.42 ± 6.01		
c.Breast	45.10 ± 6.00	0.043^*^	c > e		27.37 ± 4.75	0.004^**^	c > e		52.90 ± 7.17		
d.Liver	43.00 ± 7.26				23.56 ± 5.75				50.56 ± 8.92		
e.Head & neck	39.58 ± 4.80				21.63 ± 5.65				48.95 ± 8.71		
f.Other	42.92 ± 4.80				24.46 ± 4.35				53.11 ± 8.48		
Stage		3.105/0.080				0.020/0.886				0.404/0.526	
a.III	43.63 ± 5.68				24.64 ± 4.90				52.33 ± 8.14		
b.IV	42.06 ± 5.54				24.52 ± 4.80				51.51 ± 8.00		
Duration of diagnosis		0.654/0.521				1.688/0.188				0.760/0.469	
a.1-12months	42.14 ± 6.27				24.61 ± 4.80				51.50 ± 7.80		
b.13–24 months	43.16 ± 5.34				23.04 ± 5.20				50.60 ± 8.31		
c.>24months	43.13 ± 4.83				25.13 ± 4.65				52.75 ± 8.28		

* *p* < 0.05; ** *p* < 0.01; ***p < 0.001

In a subsequent analysis, only those significantly correlated variables were included for simultaneous regression analyses using “ACP-decisional balance” as the dependent variable (DV). As shown in Table 4 were three independent variables emerged accounting for nearly 5% of the variance (Adjusted R2 = 0.051). Then, by using “ACP-attitudes” as the DV, the three independent variable’s coefficients of variance accounted for adjusted R^2^ of 0.070. Education level and gender were significant predictors of ACP-decisional balance and ACP-attitudes; i.e., those with the higher education level (both *p* < 0.05), and females (*p* < 0.05 & < 0.01) reported more positive decisional balance and attitudes of ACP. However, cancer diagnosis on decisional balance and attitudes of ACP was not significant.

**Table 4:** The results of statistical tests for correlates of decisional balance, attitudes, and practice behaviors of ACP (*n* = 166)

	Unstandardized Coefficients						Collinearity		
Dependent Variable	*B*	SE	Beta	*t*	*p*		Tolerance		VIF
*Decisional Balance of ACP*									
(constant)	17.269	3.168		5.452	< 0.001^***^				
Attitudes of ACP	0.426	0.076	0.365	5.602	< 0.001^***^		0.907		1.102
Practice behaviors of ACP	0.293	0.044	0.418	6.612	< 0.001^***^		0.964		1.038
Gender	-0.081	0.729	0.071	-1.099	0.273		0.916		1.092
Education	0.557	0.476	0.075	1.171	0.243		0.935		1.070
Diagnosis	0.030	0.183	0.011	0.165	0.165		0.934		1.071
*Attitudes of ACP*									
(constant)	13.292	3.105		4.281	< 0.001^***^				
Decisional balance of ACP	0.385	0.069	0.449	5.602	< 0.001^***^		0.738		1.356
Practice behaviors of ACP	-0.073	0.047	-0.122	-1.547	0.124		0.768		1.302
Gender	-1.389	0.686	-0.144	-2.024	0.045^*^		0.932		1.073
Education	0.682	0.451	0.107	1.513	0.132		0.940		1.064
Diagnosis	-0.075	0.174	-0.031	-0.432	0.667		0.935		1.070
*Practice Behaviors of ACP*									
(constant)	25.090	4.356		5.760		< 0.001^***^			
Decisional balance of ACP	.748	0.109	0.524	6.858		< 0.001^***^		0.808	1.238
Attitudes of ACP	-0.211	0.127	-0.126	-1.655		0.100		0.808	1.238
* *p* < 0.05; ** *p* < 0.01; ***p < 0.001									

* *p* < 0.05; ** *p* < 0.01; ***p < 0.001

Then, by using “ACP-practice behaviors” as the DV, it was found that the coefficient of the other two independent variables accounting for adjusted R^2^ of 0.223. The accounted for variance was primarily related to ACP-decisional balance (*p* < 0.001); however, the influence of ACP-attitudes on “ACP-practice behaviors” was not significant (Table 4).

### Qualitative responses to current medical decisions or future concerns about ACP

Based upon the sub-sample’s qualitative responses to their cancer-related medical decisions and its difficulties, six themes were identified including ”compliance with physician instructions”, “family engagement in treatment decision-making”, “insufficient knowledge of the disease”, and “concerns regarding cancer treatment costs”. In addition, eight themes about future ACP-related decisions and concerns, were identified including “patients’ awareness of the Patient Right to Autonomy Act”, “family conflict”, “effectiveness of time-limited trials”, and “concerns of physician acting contrary to patients’ wishes” (Table 5).

**Table 5:** Emergent themes and selected quotations of qualitative responses (*n* = 32)

Question	Theme/ Subtheme	Selected Quotations of Respondents
What decisions have you made to treat cancer?	Decision-making method	
	1. Compliance with physician’s instructions	*We tend to do what the doctors say… We don’t argue with them… We just follow the doctors’ orders*. (#32)
	2. Independently making the decision	*My words carry weight for my family and friends. When I decided not to undergo traditional therapy, almost everyone thought I was being unreasonable. But because I’m quite bossy, I often have the final say*. (#3)
	3. Family engagement in treatment decision-making	*My mom told me to take herbals, something like South African leaf, which is super bitter. She insisted that I eat the raw leaf. The taste was terrible. She said it was to prevent disease progressed…I thought, “Why do I need to eat something to prevent it?” I didn’t want to eat it, but she forced me.* (#1). *The main reason was that my daughter insisted [that I receive percutaneous endoscopic gastrostomy (PEG)] because her colleague had the same problem as me. Her colleague hadn’t undergone PEG. The cancer was cured and he was discharged from the hospital, but he was sent to the emergency department the next day and was found to have a stomach ulcer. He had to get nutrient injections because he couldn’t eat anything. His stomach was empty, the stomach muscles kept contracting, and the stomach ulcer occurred. So, she [daughter] insisted that I undergo PEG.* (#6)
What was the most difficult decision you made?	Decision-making predicaments	
	1. Insufficient knowledge of the disease	*The doctor didn’t tell me about my illness in detail to help me understand, but I didn’t know how to ask. And then…I didn’t know how to prepare, how long would I live, what things should I leave to my family, what documents I had to prepare…The doctor didn’t tell me anything*. (#28)
	2. Concerns regarding cancer treatment costs	*It [cancer treatment] would only place more burden on my family and children. I would rather die than suffer [from the illness]. Isn’t it better for everyone?* (#6) *If your illness is so serious that it cannot be cured, medication can only make you die more slowly. Like… if you can live for 6 more months, let’s say, you need a one-on-one caregiver that costs [NT]$60,000 a month. Then, 6 × 6 = 36, you need $360,000 in total. And you need to eat, which may cost you an extra $200,000 or $500,000. Do you have enough money to cover all the expenses?* (#5)
	3. Impact on body image	*The doctor only gave me two choices. First, remove it [the bladder] and then get chemotherapy. I thought, “Is my condition that bad?” The doctor said that lots of people who wear a urine bag have lived for a long time. But this would be very inconvenient to me. Wearing a urine bag when going abroad and then coming back is quite inconvenient. It would change my entire work pattern.* (#11) *If possible, I don’t want to have chemotherapy. Having chemotherapy has weakened my immune system. I can’t hang out with friends, I keep losing my hair, and I have no energy at all. Leading such a life is meaningless. I’m not normal, and I can’t accept it.* (#28)
If you can prepare for a critical treatment decision in advance, what would you do?	Willingness to engage in ACP in the future	
	1. Willingness to make an advance directive for hospice and palliative care	*Actually, I wanted to have that marked on my NHI card. Yeah, the hospice care [making a note of hospice care order on the NHI card].* (#23)
	2. Patients’ awareness of the Patient Right to Autonomy Act	*In the first or second year when I got cancer, I started to pay attention to such news [drafting Patient Right to Autonomy Act]. When the bill was passed, I kept searching for available hospitals and visited them [to sign an advance directive], no matter which hospital, because this [ACP] is what I want.* (#11)
	3. Growing acceptance of euthanasia after long-term persistence of disease	*I guess everyone has the right to bodily integrity and autonomy. I think euthanasia can actually be a good solution because at least I can die with integrity. It’s just saying goodbye to this world, not the end of life. From a religious perspective, it [death] represents the beginning of a new life journey.* (#19)
	4. Indecision because of non-urgency and misunderstanding of decision-making timeline	*I don’t know if I can be that determined when I face it. To humans, fear and anxiety is inevitable when death is near. People and their families naturally panic when they sign the not-to-resuscitate order. In fact, I guess signing it would be a good choice…at least I can die in peace. If I talk about it [making an ACP decision] with my family now, they might think that there must be something wrong with me.* (#27)
What is the most challenging part when making such a treatment decision?	Predicaments related to advanced directive decisions in the future	
	1. Family conflict	*I remember clearly that once the doctor asked me, “No intubation? Then how about an NG (nasogastric) tube for feeding?” “Feeding is necessary, I can’t let my dad die from hunger,” I continued, “but we don’t want things like ventilator or first aid.” However, my younger brother and his wife came and stopped the doctor from practicing the DNR order to my dad for no reason. The doctor was furious, grumbling about why our family hadn’t reached an agreement beforehand. They [younger brother and his wife] strongly opposed our ACP decision. Later I learned that although our family had signed the DNR order, it might have made things difficult for the doctors if any of the family members were to oppose the decision.* (#3)
	2. Effectiveness of time-limited trials	*If the cancer I have is…irreversible, I wouldn’t want intubation. If any accident were to happen and the doctor said that intubation could save my life, then intubation would be fine. However, if the doctor expected that I would be in a permanent vegetative state even after being saved, I wouldn’t want to be rescued. Being bedridden for a long time can only burden my family, which I don’t want.* (#32)
	3. Concerns of physician acting contrary to patients’ wishes	*My grandma died painfully after being hospitalized for a long time. Every time when her heart weakened, the doctor would ask, “Do you want me to give her cardiotonic drugs?” We said “yes” after some discussion, but other indices dropped after she took the drug. Then the doctor would ask whether we want to continue this treatment because it seemed that none of the treatments worked. This process repeated several times. The doctors might not be consistent in the treatments they administer.* (#24)
	4. Fear of making a decision because of the high ACP consulting cost	*When my wife told me about it [the Patient Right to Autonomy Act], I agreed with the ideas in the act. If you’re dying, you have to let go. Don’t let the disease destroy the entire family. My wife is no longer young, she doesn’t have enough energy to take care of me. That can cost [NT] $3,000 [ACP consulting costs $3,000 per session as specified in the Patient Right to Autonomy Act], which I don’t want to pay for. The government should cover such expenses…it makes no sense to make citizens pay for it.* (#22)

## Discussion

This was apparently the first descriptive study employing a mixed-methods quantitative and qualitative design exploring self-reported ACP’s decisional balance, attitude, and practice behavior survey replies in a sample (*n* = 166) of Taiwanese patients with advanced cancer. Thus this investigation adds to the existing body of research results obtained mostly from Western cancer patients and enhances our trans-cultural understanding of ACP. Overall, the current Taiwanese advanced cancer patient sample concurred strongly with ACP as measured with DAP-ACP. The results of our investigation also revealed that gender, educational level, and cancer diagnosis were all associated with differences on both decisional balance and attitudes scales of ACP. Yet, only gender and educational level were significant predictors for both decisional balance and attitudes scales of ACP, whereas the ACP-decisioinal balance was the predictor for the ACP-practice behaviors, but not for the samples’ ACP-attitudes. The qualitative narratives that emerged from our subsample about their current medical decision included the following themes: “listen to physician instructions”, “decide the direction of treatment by yourself”, “family participation in medical decision-making”; whereas their narrative considerations of future advance directives included “pre-medical decision will”, “recognize the ACP”, severe diseases, life is not as good as “euthanasia”, “undecided” because there was no urgency and difficult to grasp at the current decision point of time. Based upon the sub-sample’s qualitative responses to their cancer-related medical decisions and its difficulties, six themes were identified including ”compliance with physician instructions”, “family engagement in treatment decision-making”, “insufficient knowledge of the disease”, and “concerns regarding cancer treatment costs”. In addition, eight themes about future ACP-related decisions and concerns, were identified including “patients’ awareness of the Patient Right to Autonomy Act”, “family conflict”, “effectiveness of time-limited trials”, and “concerns of physician acting contrary to patients’ wishes”.

Both the replies of our sample on the decisional balance and attitude scales of ACP in our participants were significantly associated, but slightly less so than that previously reported by Sudore et al. [31]. This difference may be explained by the fact that the present study recruited patients with advanced cancer and these patients may be more likely to encounter uncertainties and anxiety due to EOL treatment decisions, consequently affecting their perceptions of ACP [32]. In present study, the highest rates of participants’ agreement(s) were for the following items: “I would feel better knowing I have done what I can to plan for my future” (#8) and “Planning for future medical care only makes sense for those who are much older or sicker than I am” (#19). The latter level of concurrence suggested that, in addition to allowing patients to maintain a sense of control, the participants in our study felt it was important to discuss their care plan and treatment with their families [33]. However, at least some patients in our study believed that “only older adults with severe conditions required ACP”. In contrast, the lowest level of agreement reported by the study sample was for the ACP-attitude item: “There is no need for me to do ACP because I will not be able to make my own treatment decisions” (#16), suggesting that although many participants agreed with ACP, they felt that could not make all care plans and treatment decisions on their own. This finding was also consistent with another of our findings: namely that 81.4% and 78.9% of participants reported that they could make decisions by their own and their family as well, indicating that both the patient and their family members shared the decision–makings (SDM). Similarly, this family-bond phenomena was reported in a Chinese study by Hou et al. [34], indicating that 82% of patients preferred to discuss their care decision-makings with their family members.

The study participants relies on the ACP-practice behavior scale were mostly positive, but still somewhat less positive than that reported for subjects of an ACP web-based educational program, which allowed participants to set treatment goals in an attempt to augment their ACP-practice behaviors [31]. In this current study, the highest agreement was the for the ACP-practice behavior item: “I think of myself as someone who can reduce suffering for me and my family by doing ACP” (#31) confirming prior ACP research [35]. This finding suggested that ACP can lighten the burden on patients’ families and strengthen patients’ ties with their loved ones. The lowest scores in our participant sample were for the practice behavior items “I looked for information on ACP” (#20) and “I reviewed my advanced care documents so that I know what they say” (#23). Further, both of these items negatively correlated with ACP-practice behaviors. This suggested that most of our study participants rarely inquired about ACP and that few healthcare professionals provided relevant information. This finding was consistent with prior studies [9–11] which documented that patients with terminal diseases or long-term care residnets were willing to discuss ACP but, in fact, their medical providers rarely discussed this topic with their patients. This might due to the fact that medical personnel were reticent to broach this topic reflecting their concern that early implementation of ACP could engender fear in patients or communicate a sense of hopelessness regarding their prognosis [36]. The above evidence suggests, however, the provision of appropriate education and training for health care providers [37] and ACP-related information to patients [31] would actually serve to facilitate the dialogue of ACP between patients and their health care providers, thereby promoting practice behaviors of ACP.

In terms of the correlates of the ACP-decisional balance comprised of ACP-attitudes and practice behaviors, data from the study sample indicated that patients with preferable decisional balance (pros of ACP were superior to cons of ACP) were more likely to endorse positive ACP-practice behaviors. Other investigators have speculated that this might due to patients’ lack of familiarity with the concept of ACP [38], the level of decisional balance can reflect a patient’s evaluation of ACP [25]. In addition, the current study found that female gender and patients with higher levels of education were more likely to concur with items pertaining to decisional balance and attitudes of ACP. This latter finding was consistent with the results of Ivo et al. [39] and Miyashita et al. [40] which documented that the decision of ACP was significant related to a patient’s educational level; i.e., patients with higher educational level were more likely to report to positive behaviors on ACP. This result was consistent with the findings of Seifart et al. [41] who reported that men tended to avoid discussing death when they were worried about their last days of life; but was inconsistent with the findings of a systematic review conducted by Gadebusch Bondio et al. [42]. Additionally, our study found that the cancer diagnosis was correlated to ACP decisional balances and attitudes, indicating that patients with breast cancer tended to be embrace decision balance and attitudes toward to ACP compared to patients with head and neck cancer. We hypothesized the above difference might be primarily due to a gender confound, rather the cancer diagnosis per sec, because 89.5% of patients with head and neck cancer were male patients and 100% of patients with breast cancer were female patients in this study. Similarly, results from a Taiwanese study documented that Taiwanese women tended to give more attention to discussing EOL issues with their family members than did male patients [43]. Yet, the potential influences of gender, educational level and cancer diagnosis on ACP decision balance were not significantly correlated, except for the attitudes and practice of ACP, after the multiple regression analyses. Further, there was no statistically significant correlation between ACP-attitudes and ACP-practice behaviors in the current study. Similar to previously published studies, we inferred that ACP-practice behaviors might be potentially affected by culture, medical environment, and socioeconomic status [39]; thus, attitudes or beliefs would did not fully predict ACP-practice behaviors [44]. Also, our study determined that the age and religion of our participants were not correlated with ACP, but such findings were inconsistent results from other Asian population studies [43, 45, 46]. For example, studies conducted in Hong-Kong [45], Korean [46], and Taiwan [43] found that older patients (age above 50 or 70 years old) had higher acceptance or readiness to receive palliative care [45] and AD or ACP [43, 46]. Only one third (32%) of participants in this current study were old patients perhaps contributing to our inability to detect the age differences on the ACP. Simliar to a Taiwanese study by Chan et al. [43], we failed to find any significant correlation between participants’ religion and their replies on the ACP. It might be due to what the role of religion playing in the perception of ACP for patients; i.e. a systematic review (*n* = 36) of Asian patients’ perspectives on ACP [47] found that religion perhaps served as facilitors to motivate patients to engage in ACP, but the region might serve as barriers if the perception of ACP was inconsistent with their religion beliefs [25].

By comparing the quantitative and qualitative findings revealed that the most consistent comparable result was with the narrative theme “compliance with physician instructions” (qualitative response) and the APC item: “There is no need to do ACP because my doctor knows what I want for my EOL care” (#15). These finding confirmed similar findings from other studies, especially those conducted in China [48] and Taiwan [9], implying that physicians are granted a great deal of authority, which apparently trumps patients attitudes and wishes in some circumstances. Somewhat paradoxically, some of the current study’s qualitative findings were not consistent with quantitative findings from the same sample. For instance, participants’ ACP-related decision-making could also be influenced by their “insufficient understanding of their illnesses”, “concerns regarding the cost of treatment”, and “the impact on body image”. The results were, however, similar to prior quantitative studies documenting cancer patients’ expressed desire to know the consequences of their diseases [49]. Yet, research suggests that physicians were reluctant to discuss prognosis with their patients for fear of potentially adversely affecting their patients’ mental health [50, 51]. Also, cancer can impose heavy financial burdens on patients due to the high costs of medical care and nutrition supplements as well as their inability to work [52, 53].

The major concerns emerging in the qualitative results regarding advanced cancer patients’ ACP-related decisions included “family conflict,” “effectiveness of time-limited trials,” and “concerns over the physician going against patients’ self-will”. These qualitative findings surfaced several factors leading to EOL experiences, such as avoidance of futile treatment at near-death stage, alleviation of burden to family, and strengthening of ties with loved ones [35]. Due to the concern of patients’ rights in medical decision-making, the participants’ narratives in this study reflected their expressed concerns about their medical teams considerations to timely initiate “time-limited trials”. The latter referring to the implementation of medical interventions for a limited time period to achieve a specific goal (i.e., if the patient’s condition improves, the intervention continues; if the patient’s condition worsens, the intervention is terminated) [54], to improve the quality of their ACP decisions. In interpretating our findings one needs to be cognizant of several limitations of the present study. Firstly, the study sample was purposely drawn from a regional teaching hospital in the southern Taiwan and consisted of a relatively small sample (*n* = 166) with nearly 40% of eligible patients declining to participate (*n* = 109). This may limit the generalizability of our findings due to the questionable representativeness of our sample. Secondly, the practice behaviors of ACP in this study were primarily measured with a 15-item subscale of DAP-ACP that was more likely to describe the intention or tendency of to undertake ACP practice behaviors, rather than actual ACP behavioral changes. Lastly, nearly 80% of patients in this study claimed that their family members shared their related medial care decision-makings; yet we did not include patients family members, thus the potential influences of family members on patients’ACP replies could be ignored.

## Conclusions & Recommendations

This study showed that participants concurred, that is, mostly patients with advanced cancer agreed with and/or intend to participate in ACP process. In addition, the participants’ gender and education level predicted both the decisional balance and attitudes of ACP and the participants replies on the ACP-practice behaviors were correlated with the ACP-decisional balance replies, but not for the ACP-attitude. Analyses of the qualitative responses revealed various themes that emerged were consistent with the majority of those documented by the quantitative results, such as compliance with physician instructions. However, several factors influencing the participants’ ACP-related decisions identified in the qualitative responses were not assessed by the DAP-ACP measure, such as insufficient understanding of their illnesses. This is important to note since studies of such highly personal-related experiences, ACP issue, mixed methodology designs may shed additional light on this patient domain yielding a broader cultural and person/patient-oriented perspectives.

One recommendation for future research is to intiate a multi-centre design to enroll not only larger number of patients, but also sample subjects from a number of rural and urban centers in an effort to improve the generalizability of study findings. Secondly, future studies incorporate an external criterion outcome validation measure; for instance, whether participants signed the legal ACP related document during the study period to allow the researcher further examinations of the casual correlations and directionality among decisional balance, attitudes and practice behavior of ACP. Thirdly, we suggest future studies with a longitudinal design and follow up for a extended period of time to capture the participants’ concurrent measures of balance, attitudes, and practice of ACP. Lastly, the influences of family members on patients’s process of ACP decision-making ought to be taken into account by including family members (dyads) in future studies, to reflect the family-bond phenomena in the Chinese culture. The results from our study support nascent efforts to educate health providers in ACP-related knowledge, to strengthen their communication skills, to provide the ACP-knowledge and skill-set needed to educate patients and their families, and to embrace a multidisciplinary approach–thus ensuring efficient ACP-related decision-making [55].

## Electronic supplementary material

Below is the link to the electronic supplementary material.

Supplementary Material 1

## Data Availability

Prior to consenting to participate in the study, all potentially eligible patients were informed with that their interview responses and provided information would be stored confidentially and only could accessed by the first author. The datasets used and analyzed during the current study are available from the corresponding author on reasonable request.
