# Supplementary material for: The decisional balance, attitudes, and practice behaviors, its predicting factors, and related experiences of advance care planning in Taiwanese patients with advanced cancer
Source: BMC Palliat Care. 2022 Nov 2;21:189. doi: 10.1186/s12904-022-01073-5 (PMC9628122; doi:10.1186/s12904-022-01073-5)
Supplement: Supplementary file 1 — Supplementary Material 1 [file 12904_2022_1073_MOESM1_ESM.docx]

Appendix 1. The exploratory factor analyses (EFA) of decisional balance, attitudes, practice behaviors of Advance Care Planning (ACP) (*n* = 166)

| Item | Factor 1 | Factor 2 | Factor 3 |
| --- | --- | --- | --- |
| **ACP-decision balance subscale (12 items)** |  |  |  |
| 1.It would be hard to do ACP because I don’t like thinking about being very ill. | .769 |  |  |
| 2.Doing ACP would simplify how decisions would be made if I were very ill. |  | .332 |  |
| 3.It would be hard to do ACP because I don’t like thinking about death. | .866 |  |  |
| 4.I don’t want to talk with loved ones about end-of-life decisions. | .648 |  |  |
| 5.Doing ACP would make it easier on my close family and friends. |  | .712 |  |
| 6.It would be hard to do ACP because there are too many options to consider for my end-of-life care. | .835 |  |  |
| 7.Understanding my wishes would help my loved ones to ensure I get the care I want. |  | .567 |  |
| 8.I would feel better knowing I have done what I can to plan for my future. |  | .762 |  |
| 9.ACP would go against my lifestyle of living in the moment. | .642 |  |  |
| 10.Doing ACP would give me peace of mind. |  | .813 |  |
| 11.ACP would help me to keep control over what happens to me at the end of life. |  | .690 |  |
| 12.It doesn’t make sense to do ACP because my wishes for my end-of-life care might change. | .533 |  |  |
| **ACP-attitudes subscale (7 items)** |  |  |  |
| 13.If you fill out a document such as a living will, the doctors are more likely to stop life support too soon. | .674 |  |  |
| 14.There is no need for me to do ACP because once you reach a certain age; the doctors aren’t going to use machines to try to keep you alive. | .751 |  |  |
| 15.There is no need to do ACP because my doctor knows what I want for my end-of-life care. | .693 |  |  |
| 16.There is no need for me to do ACP because I will always be able to make my own treatment decisions. | .821 |  |  |
| 17.ACP would interfere with the plans that the Lord has for me. | .730 |  |  |
| 18.There is no need for me to do ACP because if I am made to suffer, then there must be a good reason for it. | .699 |  |  |
| 19.Planning for future medical care only makes sense for those who are much older or sicker than I am. | .692 |  |  |
| **ACP-practice subscale (15 items)** |  |  |  |
| 20.I looked for information on ACP. |  | .860 |  |
| 21.I thought about information people have given me on ACP. |  | .811 |  |
| 22.I remembered information people have given me on the need for ACP. |  | .855 |  |
| 23.I reviewed my advanced care documents so that I know what they say. |  | .538 |  |
| 24.There is someone I can talk to about doing ACP. |  |  | .712 |
| 25.It is important that I make sure people close to me have copies of my advanced care plans with them. |  |  | .644 |
| 26.Now is the right time to do ACP. | .630 |  |  |
| 27.It is important that I make sure that I know where my advanced care documents can be found. | .639 |  |  |
| 28.I can do ACP even if it is difficult for my loved ones. | .702 |  |  |
| 29.Doing ACP makes me feel like a person who cares about my close family and friends. | .784 |  |  |
| 30.I can count on my loved ones to help me with ACP. |  |  | .610 |
| 31.I think of myself as someone who can reduce suffering for me and my family by doing ACP. | .776 |  |  |
| 32.My loved ones will support me as I do ACP. | .680 |  |  |
| 33.The thought of having an advanced care plan makes me feel good about taking responsibility for my health care. | .783 |  |  |
| 34.I feel committed to doing ACP. | .812 |  |  |
